# The contribution of education-specific mortality trends to the life expectancy stagnation in England & Wales

**DOI:** 10.1007/s10654-025-01251-8

**Published:** 2025-06-02

**Authors:** Jesús-Daniel Zazueta-Borboa, Leo van Wissen, Alison Sizer, Fanny Janssen

**Affiliations:** 1 Netherlands Interdisciplinary Demographic Institute, KNAW/University of Groningen, Lange Houtstraat 19, The Hague, 2511 CV The Netherlands; 2 Population Research Centre, Faculty of Spatial Sciences, University of Groningen, Groningen, The Netherlands; 3 Department of Information Studies, University College London, London, UK

**Keywords:** Mortality, Population health, Stagnation, England & Wales, Life expectancy, Educational inequalities

## Abstract

The increase in life expectancy at birth (e0) has stagnated since 2011 in England & Wales (E&W). Prior research hypothesized that the stagnation is related to increasing austerity measures and widening socio-economic inequalities. We formally assessed the contribution of education-specific mortality trends and increasing educational inequalities to the stagnation. We used individually-linked mortality data by sex, educational attainment (low, middle, high), and age (30+) from the ONS Longitudinal Study. We compared, by sex, the observed with the expected (= projected) increase in remaining life expectancy at age 30 (e30) in 2011–2017 for the national and education-specific populations. We assessed the education-specific mortality contributions using stepwise decomposition, and the contribution of increasing educational inequalities using a scenario analysis that assumes constant inequalities. In E&W in 2011–2017, e30 increased by 1.32 years (males) and 1.14 years (females) less than expected, which translates into 2.3 and 1.9 months annually. Stagnation of the increase in e30 occurred across all educational groups, with declines in e30 after 2011 for middle-educated males and low-educated females. Mortality trends among low-educated males (41.4%), middle-educated males (53.5%), and low-educated females (86.1%) contributed the most to the sex-specific stagnation. The observed increases in educational inequalities between 2011 and 2017 contributed approximately 27% to the national e30 stagnation.Widening educational inequalities, and particularly the unfavourable mortality trends observed for middle educated males and low educated females since 2011, contributed substantially to the e30 stagnation since 2011 in England & Wales.

**Supplementary Information:**

The online version contains supplementary material available at 10.1007/s10654-025-01251-8.

## Introduction

Across high-income countries, progress in life expectancy at birth (e0)– which is an important summary measure of population health– has slowed since 2010 [1]. In England & Wales, the slowdown in improvements in e0 was much steeper than in other high-income countries, except for the United States of America [2],

Previous research highlighted that this stagnation is not linked to a potential maximum level of life expectancy attained, and that it is related to less favorable mortality trends since 2011 at all ages, but particularly below age 50 [2, 3]. Moreover, the stagnation is related to increases in causes of death such as winter influenza, cardiovascular diseases, and preventable and treatable cancer among working-age groups [4].Furthermore, previous research hypothesized the potential role of increases in austerity measures, and, relatedly, of increases in socio-economic mortality and health inequalities [5, 6]. However, no previous study has formally assessed the contribution of mortality by socio-economic group nor of increasing socio-economic mortality inequalities on the life expectancy stagnation in England and Wales. It seems likely that increases in socio-economic mortality and health inequalities contributed to this stagnation. Previous analysis showed that the increases in austerity measures, such as reductions in local government spending and in social security among the working-age population, were correlated with e0 losses in the 2011–2017 period [7, 8]. Furthermore, progress in e0 has been more modest in the most deprived socio-economic areas of England than in the less deprived areas [5, 6]. In addition, previous literature has pointed out that mortality below age 50 has been important in explaining the observed widening of life expectancy inequalities in England & Wales [9], and that the abovementioned causes of death have increased more in areas of England & Wales with higher rather than with lower socio-economic deprivation levels [5].

We formally assess the contribution of education-specific mortality trends and of increasing educational inequalities on the observed stagnation in life expectancy in England & Wales between 2011 and 2017. In doing so, we (i) propose a comparative method to measure (yearly) stagnation (ii) apply advanced demographic techniques; and (iii) use individually-linked mortality data by educational level, rather than the area-level socio-economic information used by previous studies of socio-economic mortality inequalities in the United Kingdom [5, 10].

### Data and methods

We used individually-linked mortality data from the Office for National Statistics Longitudinal Study (ONS-LS), which contains census and life events data beginning in 1971 for a 1% representative sample of the England & Wales population [11]. We transformed the data to annual mortality by educational attainment group (low, middle, or high), single age (30, 31, 32… 100), and sex from 1971 to 2017, while adjusting for data issues, including differences with country-level morality data [12].

We obtained the remaining life expectancy at age 30 (e30) by sex for both the national population and the three educational groups for the years 1971–2017 by applying standard demographic life table techniques to the underlying age-specific mortality rates [13]. To identify the start year of the stagnation in e30 (2011), and to assess the study period (1999–2017) we examined trend breaks in the sex-specific trends in the national-level e30 (1972–2017) by applying segmented regression [14].

We measured, by sex, the e30 stagnation as the difference between the observed and the expected increase in e30 between 2011 and 2017, in keeping with the benchmark method to obtain the mortality impact of COVID 19 [15]. The expected increase in e30 was based on a Lee-Carter projection [16] of age-specific mortality improvements over the 1999–2011 period.

To assess the contribution of education-specific mortality to the stagnation in e30, we decomposed, by sex, the differences between the observed and the expected change in e30 over the 2011–2017 period using stepwise replacement decomposition [17]. The inputs for our decomposition were the observed and the expected age-specific mortality rates and population sizes by educational group and sex.

We assessed the contribution of increasing educational inequalities to stagnation in e30 by a scenario analysis in which we estimated the level of stagnation if the average level of educational inequalities in 2000–2010 (e30 high educated minus e30 low educated; e30 high educated minus e30 middle educated) pertained in 2011–2017.

For more detailed information regarding the data and methods, and a sensitivity analysis, see Supplementary File 1.

## Results

**Fig. 1:**
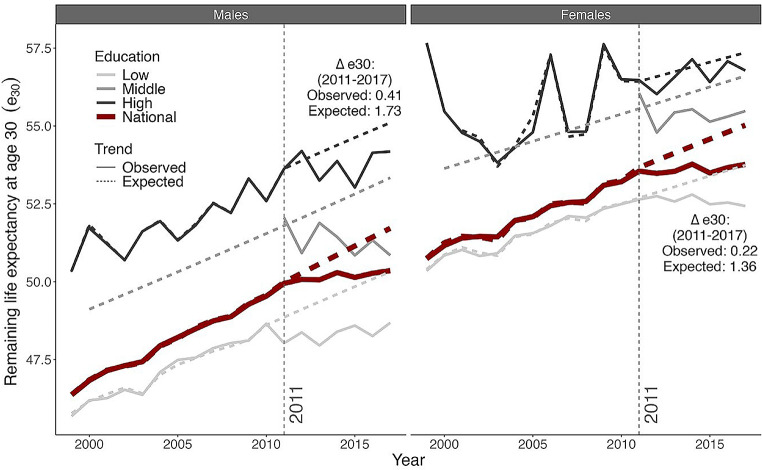
Observed (1999–2017)* and expected (2011–2017)** trends in remaining life expectancy at age 30 (e30), by sex, in England & Wales. At the national level and across educational attainment groups. The vertical dashed lines represent the start of the stagnation in e30. The observed and expected values correspond to National e30 * See Supplementary File 1 - Figure S3 for a similar figure that displays the observed trends from 1971 to 2017. ** To obtain the expected e30 trends, we applied a Lee-Carter projection to age-specific mortality improvements from 1990 to 2011. For middle-educated males and middle-educated females, we however instead obtained the expected e30 values based on the projection of mortality improvements over the 1972-2000 period. We did this because the e30 values for the middle-educated in 2002-2010 were substantially lower than the historical trend (see Figure S6 in Supplementary File 1) which could potentially be the result of data issues in the 2001-2011 follow-up (see the data section of Supplementary File 1)”. Source data: ONS Longitudinal Study.

The observed increase in e30 in England & Wales stagnated after 2011, not only at the national level, but also across all educational groups (Fig. 1). If mortality conditions from 1999 to 2011 had continued into the 2011–2017 period, e30 would have increased by 1.73 years rather than by the 0.41 years observed for males, and by 1.36 years rather than by the 0.22 years observed for females (Fig. 1A). Consequently, at the national level, e30 increased by 1.32 years (males) and 1.14 years (females) less than expected, which translates into an annual stagnation of 2.28 and 1.92 months, respectively (Supplementary File 2: Figure S1).

For the low-, middle-, and high-educated, the differences between the expected and the observed increases in e30 between 2011 and 2017 (Fig. 1B) amounted to 0.81, 2.75, and 0.91 years, respectively, for males and to 1.26, 1.12, and 0.61 years, respectively, for females. See Supplementary File 2: Figure S1 for the respective annual stagnation expressed in months.

**Fig. 2:**
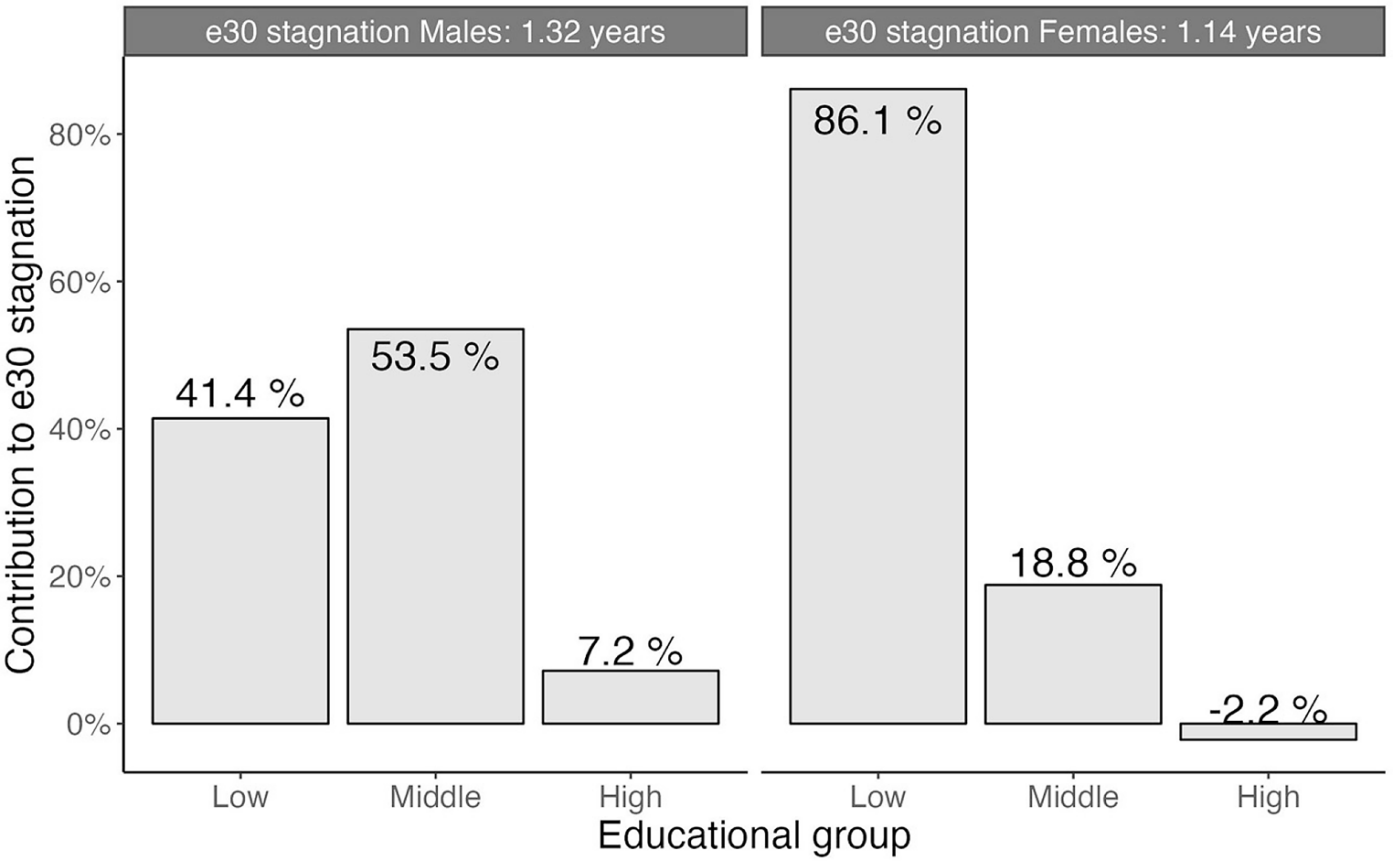
Contribution of education-specific mortality trends to the observed e30 stagnation in e30 from 2011 to 2017 (1.32 years among males; 1.14 years among females), by sex, England & Wales *The abovementioned (unrounded) contributions add up to 102% for both males and females because the contributions were counterbalanced by 2% for both males and females by changes over time in the population composition by education. This 2% from population composition effect, reflects the difference in population composition contribution between observed and expected e30.Source data: ONS Longitudinal Study

For males, the contribution of education-specific mortality trends to the observed stagnation (1.32 years) was 41% for the low-educated, 54% for the middle-educated, and 7% for the high-educated (Fig. 2). For females, mortality trends among the low-, middle-, and high-educated contributed 86%, 19%, and − 2%, respectively, to the observed stagnation of 1.14 years. The gradual process of educational expansion (Supplementary File 2: Figure S2) suggests no important role of the low educated becoming more selected and more prone to mortality over time.

Educational inequalities in e30 were higher after 2011 compared to the average level in 2000–2010 (Supplementary File 2: Figure S3). Had the average inequality level remained, e30 would have increased 1.0 years (instead of 1.3 years) less than expected for males and 0.8 years (instead of 1.1 years) less than expected for females, indicating a contribution of respectively 26.5% (1–73.5%) and 27.2 (1-72.8%) of the increasing inequalities (Supplementary File 2: Figure S4 and Table S1).

## Discussion

We found that in England & Wales in 2011–2017, e30 increased by 1.32 years (males) and 1.14 years (females) less than expected, which translates into an annual stagnation of 2.3 and 1.9 months, respectively. The trends for e30 are very similar to the trends in e0 (see Supplementary File 2: Figure S5; Table S2), and, as such, our results on e30 stagnation align with the previous findings regarding the stagnation in e0 [1, 2, 4].

Our novel way to assess (annual) life expectancy stagnation– inspired by the benchmark method to quantify the mortality impact of COVID-19– enabled a comparative assessment of stagnation, including the estimation of excess deaths. We found that the stagnation in e30 accumulated to a total of 267,218 excess deaths between 2011 and 2017 (120,427 among males; 146,791 among females) (see Supplementary File 2– Figure S7). These numbers are much larger than the estimated number of excess deaths during the COVID-19 pandemic (152, 255) [18].

Our results furthermore showed that the stagnation occurred unevenly across the educational groups, and that those educational groups that experienced decreases in e30 after 2011 (middle-educated males and low-educated females) contributed the most to the observed stagnation. These decreases in e30 were due to a deterioration across all age groups among middle-educated males, while for low-educated females (and males) they were due to unfavourable mortality trends below age 49, at ages 60–69 (females) and 90+ (see Supplementary File 2: Figure S6).

The resulting observed increases in educational inequalities in e30 since 2011 in England & Wales contributed substantially (around 27%) to the observed life expectancy stagnation, and it was stronger than in other countries for which this was recently studied (Finland; Italy (Turin) [9]) but smaller than in the USA where life expectancy has also stagnated since 2010 [19]. These results, coupled with the evidence from previous literature, point to a likely role of the introduction of austerity measures after the 2008 economic crisis in explaining the observed stagnation in e30 in England & Wales after 2011. The austerity measures affected vulnerable population groups the most [5], and (i) increased the uptake and the impact of unhealthy behavioral factors (alcohol, obesity) among the low-educated at younger ages [20] and (ii) affected health care access and treatment related to cardiovascular mortality among the low-educated at older ages [21]. These two different mechanisms align with the different age groups for which mortality particularly deteriorated for the low-educated and for the middle-educated (see before). Previous own analysis furthermore revealed important increases in educational inequalities in alcohol-attributable mortality for England &Wales as a result of– in particular– increasing alcohol-attributable mortality among the low and middle-educated in the years just before 2017 [22]. In the absence of alcohol-attributable mortality, educational inequalities in e30 in England & Wales showed a continued (declining) trend from the 1980s [22].

We recommend exploring the role of possible increasing socio-economic inequalities in the recent stalling life expectancy in other high-income countries as well, such as the USA where educational inequalities in mortality have also grown and life expectancy stagnated.

For England and Wales, our results imply that actions to (i) reduce the uneven uptake and impact of unhealthy behavioural lifestyle factors (alcohol, obesity) across educational groups, and (ii) to diminish socio-economic inequalities in (access to) care and treatment related to cardiovascular diseases, are imperative to reduce socio-economic inequalities in mortality and to sustain continued improvement in life expectancy.

## Electronic supplementary material

Below is the link to the electronic supplementary material.

Supplementary Material 1

Supplementary Material 2

## Data Availability

Data may be obtained from a third party and are not publicly available. The secondary data that we used as input for our analyses cannot be made publicly available, because the institutes that own the data apply a restricted access policy. The specific output that was used to create the main tables and the figures can be obtained through Open Science Framework (https://osf.io/37fne/). The R code(s) can be requested from the first author.
