# Supplementary material for: The contribution of education-specific mortality trends to the life expectancy stagnation in England & Wales
Source: Eur J Epidemiol. 2025 Jun 2;40(5):511–5. doi: 10.1007/s10654-025-01251-8 (PMC12170670; doi:10.1007/s10654-025-01251-8)
Supplement: Supplementary file 1 — Supplementary Material 1 [file 10654_2025_1251_MOESM1_ESM.docx]

Supplementary File 1 –

The Contribution of Education-Specific mortality trends to the life expectancy stagnation in England & Wales

SUPPLEMENTARY DATA & METHODS

**Journal:** European Journal of Epidemiology

Data

We examined all-cause mortality by the highest educational level attained, sex, and single age from age 30 to age 100 for England & Wales for the years 1972-2017. We focused on individuals aged 30 and older to ensure the validity of educational attainment as a measure of socio-economic status (SES).

For this purpose, we used data from the Office for National Statistics Longitudinal Study (ONS-LS) (ONS, 2019), which contains individual information on demographic and socio-economic variables obtained through the censuses (1971, 1981, 1991, 2001, 2011), as well as individually-linked information on life events (including births, deaths, and cancer registrations), for an approximately 1% representative sample of the population of England & Wales (Shelton et al., 2018).

For our analysis, we employed a 10-year follow-up of the ONS-LS sample members aged 20 and older at the time of the census in 1971, 1981, 1991, 2001, and 2011. We followed these individuals until the next census, or until the date that vital status information was last linked to the ONS-LS (currently 31 December 2017). We rearranged the individual-level cohort data (20+) for the five different follow-up periods (1971-1981, 1981-1991, 1991-2001, 2001-2011, 2011-2017) into aggregate period data (30+; 1972-2017). For more detailed information regarding the data used, see https://www.futurelongevitybyeducation.com/background-information/ (Password = VICI_info).

We used education as a measure of socio-economic status, because data on education are available for longer time trends, and education tends to capture the SES of females much better than income or occupation (Eikemo et al., 2014). Using the International Standard Classification of Education (ISCED) (UNESCO, 1997), we created three educational attainment groups, in line with previous international research (de Gelder et al., 2017): low (no, preprimary, primary, and lower secondary; ISCED-1997 0-2), middle (upper secondary and post-secondary non-tertiary education; ISCED-1997 3-4), and high (tertiary education; ISCED-1997 5-6).

The data for England & Wales have been adjusted to enable the use of this three-group educational structure, and to deal with trend discontinuities related to identified data issues (Janssen et al., 2024). This included the adjustment for differences with country-level mortality data for the total England & Wales population.

We dealt with (potential) missing data for educational attainment in our data selection and data handling process (see Janssen et al. 2024). First, because educational attainment information for immigrants is not available when they enter the country, we did not count immigrants until the census and the follow-up period after they arrived in England & Wales in order to ensure that we had complete information on educational attainment. Second, we carefully explored the amount of missing information on educational attainment, and consequently dealt with them (Janssen et al. 2024). By using the detailed underlying educational variables in the ONS-LS, we ended up with missing educational information for less than 0.5% of the respondents in the 1991 follow-up and the 2011 follow-up (Janssen et al. 2024 Table 1). In the 2001 follow-up, educational information was initially missing for all respondents aged 75 and older, because they were not asked about their educational level during the 2001 census. By using their educational information from the 1991 census instead, the percentage of respondents with missing educational information amounted eventually to 1% among males, and 1.3% among females. We proportionally redistributed the deaths and personyears with missing educational information to the low, middle, and high education categories according to the smoothed relative share of personyears and deaths of the respective educational category by year, sex, and age. The underlying assumption that those with missing education were missing at random is made implicitly in previous research that omitted those with missing educational information.

We carefully assessed the occurrence of (potential) data issues hampering the examination of long-term mortality trends by educational level for E&W in a time-consistent and internationally comparable manner, and adjusted the data – as much as possible - for the identified data issues (see Janssen et al. 2024). In sum, the four main data issues we identified were: 1) Inconsistent information on educational attainment over time. 2) Missing information on educational attainment 3) Inconsistent estimation of emigration 4) Imperfect alignment of the ONS-LS data selection with country-level population and mortality data for the general E&W population. Our data adjustment approach entailed the optimization of the available education information at the individual level, and the adjustment—at the aggregate level—for trend discontinuities related to the identified data issues, and for differences with country-level mortality data for the total population. Despite the meticulous effort, and the obtained consistent mortality trends by education over time for the total male and female population aged 30 and over, still a trend break in mortality appeared among males aged 30-74 (see Figure 3 in Janssen et al. 2024), which resulted in higher mortality and consequently lower e30 values for the middle-educated in 2002-2010, compared to the years before and after. Because we could not relate this remaining trend break to one particular data issue that we identified, and because the trend break only seemed to apply to the male population aged 30-74, we decided against a potential additional adjustment. However, the timing of the trend break (starting at the onset of the 2011 follow-up and ending at the end of the 2011 follow-up) seems indicative that a potential (additional) data issue could be at stake, and therefore the mortality data for the middle educated aged 30-74 should be treated with caution.

We used mortality data from the Human Mortality Database (HMD, 2021) by sex, single year of age, and calendar year to compare our results regarding the stagnation of remaining life expectancy at age 30 with those regarding the stagnation of life expectancy at birth (e0).

Methods

- Life tables

To study trends over time in remaining life expectancy at age 30 (e30) for the national population and the three educational groups, we constructed period life tables by applying standard period life table techniques (Preston et al., 2001) to age-specific mortality rates for the national population and by educational group. We computed e30, by sex, for the national population and the three educational attainment groups for each single calendar year.

Because the ONS-LS data apply to a 1% representative sample of the total E&W population and not to the full E&W population itself, the e30 estimates across education groups - particularly the middle and high education groups - come with some uncertainty. To illustrate the uncertainty of the e30 estimates we calculated 95% confidence intervals by sex, education, and year for the complete time series (Supplemental Figure 1). We obtained the confidence intervals for e30 by performing a Poisson sampling of age-specific death counts for each year, sex, and educational group. We then calculated life tables for each of the 1,000 replications and obtained the e30. This approach is based on Silcocks et al. (2001) and has been applied previously to estimate confidence intervals around life expectancy losses during the COVID-19 pandemic (Aburto et al. 2021).

**Figure S1.** Time trends in remaining life expectancy at age 30 (e30) at the national level (Panel A) and by educational attainment groups (Panel B), including 95% confidence intervals, by sex and year. England & Wales, 1972-2017. *We obtained 95% confidence intervals using 1,000 simulations of Poisson sampling of age-specific death counts.*


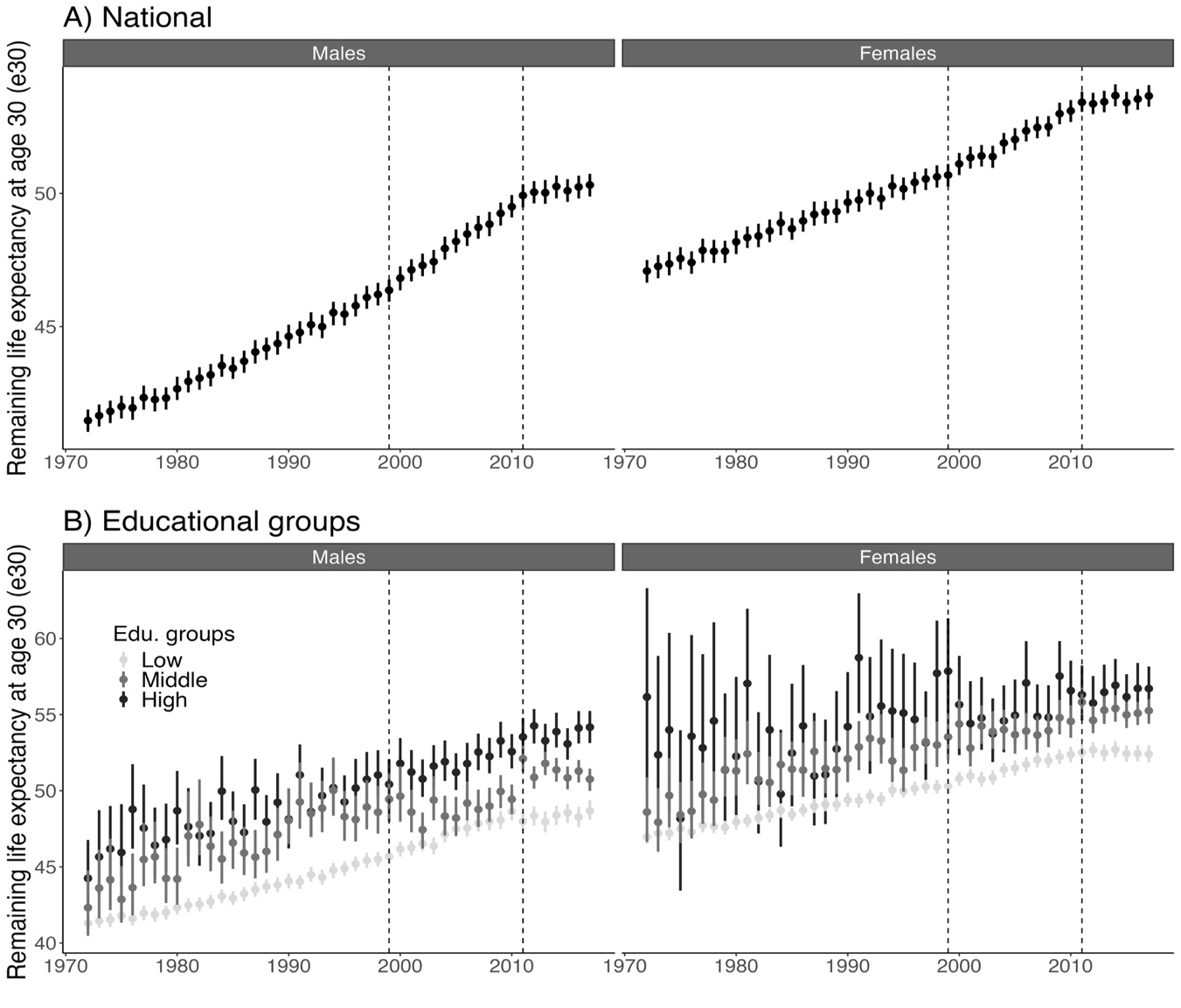


Source data: ONS-Longitudinal Study.

In addition, we smoothed the trends in e30 at the national level and by educational groups and trends in educational inequalities in e30 using segmented regression (Figure S2). We smoothed those trends using segmented regression analysis by sex and educational groups. Using segmented regression can help us, to deal with uncertainty regarding trends rather than the uncertainty in the life expectancy estimate (as the bootstrapping technique helps us to get the uncertainty in the life expectancy estimate but not on the trends). The advantage of segmented regression is that it allows us to get confidence intervals for the trends in life expectancy and educational inequities in e30.

**Figure S2.** A)Time trends in remaining life expectancy at age 30 (e30) at the national level and by educational attainment groups and B) time trends in educational inequalities in e30 (in years) by sex, including 95% confidence intervals, by sex. England & Wales, 1999-2017. *We obtained 95% confidence intervals using segmented regression analysis.*


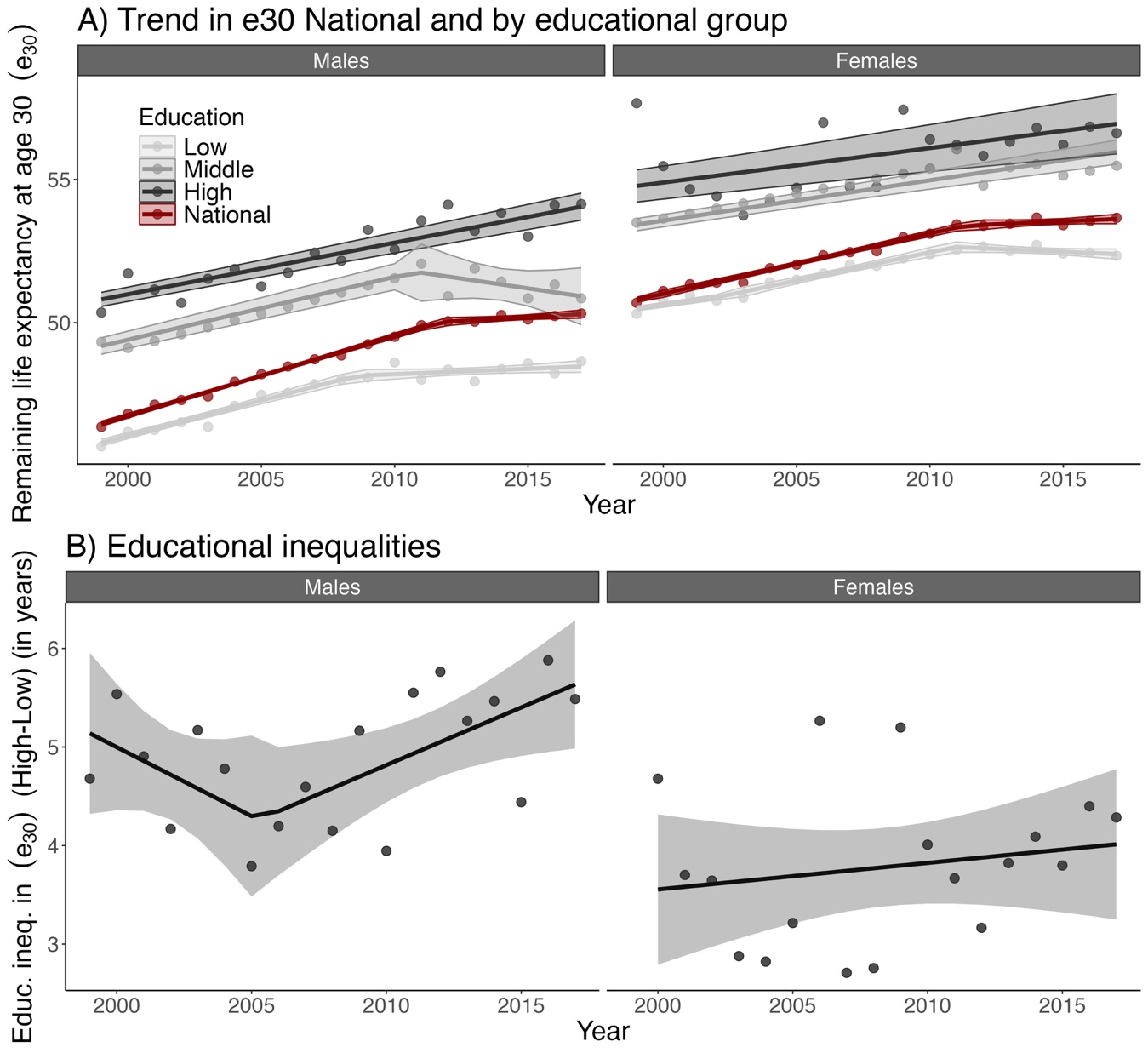


Source data: ONS-Longitudinal Study.

- Segmented regression

To identify the start year of the stagnation in e30, and to assess the study period, we examined trend breaks in the sex-specific trends in the national-level e30 (1972-2017) using segmented regression. More specifically, we employed a Davies test to assess the existence of a non-constant regression parameter in, first, a linear regression model, and, subsequently, a model with one, two, or three trend breaks. To assess whether the identified trend breaks were statistically significant (p-value of <0.05), we performed a likelihood ratio test. Our segmented regression analysis was carried out using the R package “*segmented*” (Muggeo, 2008).

From our segmented regression analysis, we identified three trend breaks in e30 for males (1979, 1998, 2011) and two for females (1999, 2011) (see Table S1 and Figure S3 below). For both males and females, e30 increased strongly between 1998/1999 and 2011, and the increase in e30 stagnated after 2011. Based on these findings, we identified the year 2011 as the starting year of the stagnation, and 1999-2017 as our study period.

**Table S1.** Phases of change in trends in remaining life expectancy at age 30 (e30) at the national level, identified by breakpoints and slopes, including (in brackets) their confidence intervals (95%), by sex, England & Wales, 1972-2017. *All trend breaks are statistically significant at p-value <0.05.*

| Sex | Break | | | Slope | | | |
| --- | --- | --- | --- | --- | --- | --- | --- |
|  | 1 | 2 | 3 | 1 | 2 | 3 | 4 |
| Males | 1979 | 1998 | 2011 | 0.13 | 0.20 | 0.28 | 0.05 |
|  | (1976 to 1981) | (1996 to 1999) | (2010 to 2012) | (0.09 to 0.16) | (0.18 to 0.21) | (0.26 to 0.29) | (0.00 to 0.09) |
| Females | 1999 | 2011 |  | 0.14 | 0.21 | 0.04 |  |
|  | (1996 to 2001) | (2010 to 2012) |  | (0.13 to 0.14) | (0.19 to 0.23) | (-0.01 to 0.09) |  |

* Note: the number in each column indicates the number of the corresponding breakpoints obtained from segmented regression analysis. For example, slope 1 is the slope between the starting point of our observation, and the first breakpoint. Slope 2 is the slope between the first and the second breakpoint.

Source data: ONS Longitudinal Study

**Figure S3.** Time trends in remaining life expectancy at age 30 (e30), by sex, in England & Wales, 1971-2017. *The dashed line represents the start of stagnation in the increase in e30, while the solid line represents additional trend breaks.*


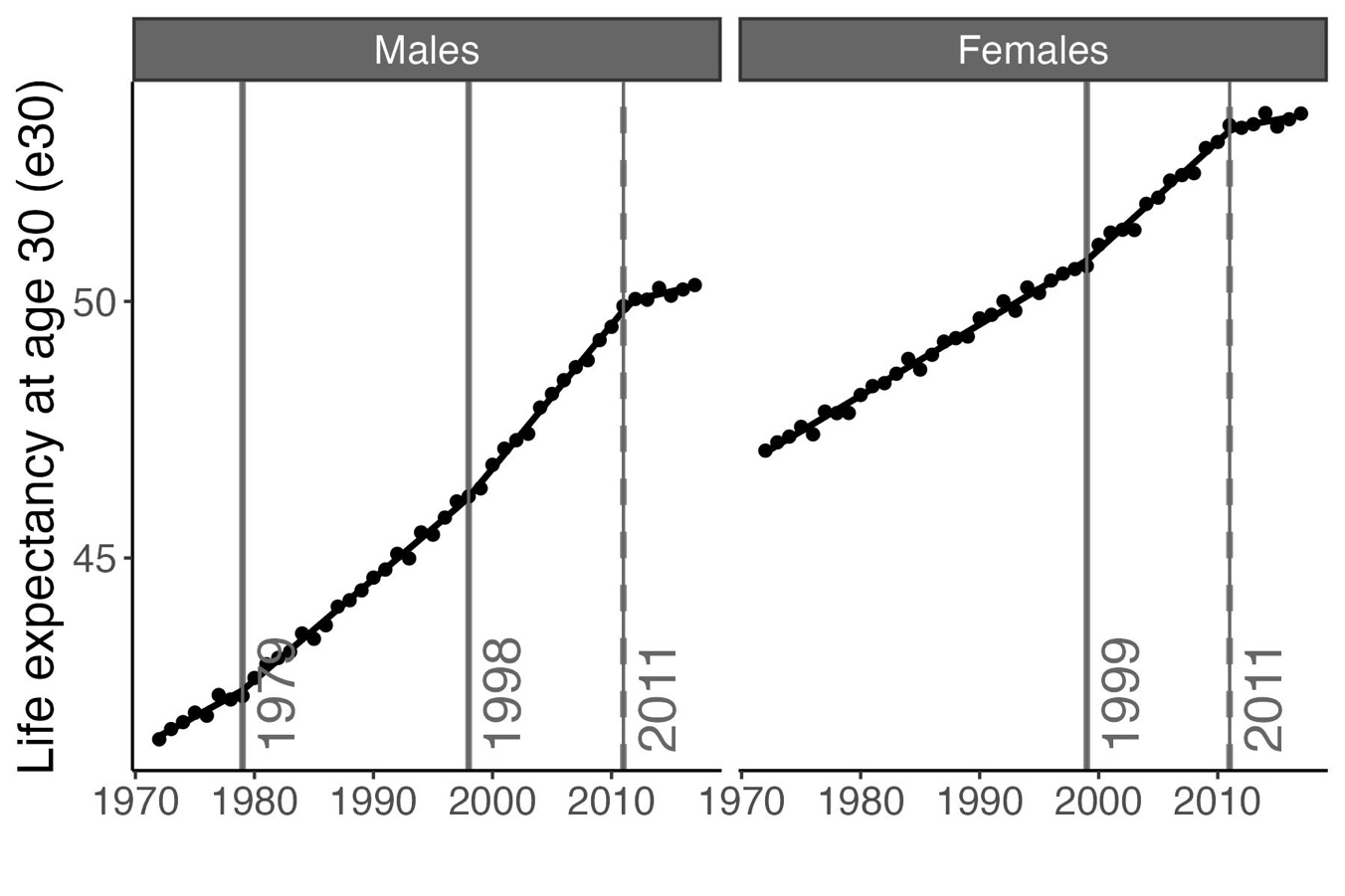


Source data: ONS Longitudinal Study

- Operationalization of e30 stagnation

With e30 stagnation we refer to the situation in which the previous increase in e30 either diminished (= slowdown), turns into stable e30 levels (reversal to stable levels), or turns into a decline (reversal to decline).

We measured stagnation in e30 between 2011 and 2017 as the difference between the observed and the expected increase in e30 between 2011 and 2017 (see the next section on how the expected increase in e30 is measured). This approach stems from the comparison of the observed with the expected deaths, which is considered the most reliable way to quantify the mortality impact of COVID-19 (Islam et al., 2021; Marinetti et al., 2023). We obtained the stagnation by subtracting the observed increase in e30 from the expected increase in e30.

In addition to the overall stagnation, we estimated the annual stagnation by dividing the overall difference by seven and expressed this in months by multiplying again by 12. Our operationalization of (annual) stagnation enables a comparative assessment of e30 stagnation, including an assessment of excess deaths, and its decomposition into mortality patterns for underlying population groups.

- Method to obtain expected e30

Previous estimates of the stagnation in e0 (e.g. Abrahms et al. 2023) purely relied on past trends in e0, for example by comparing the increase in e0 in 2011-2019 with the increase in e0 in 2000-2010, and – as such – assumed that e0 can continue to increase at the same rate as in the past. The Lee-Carter methodology does not make this assumption and relies on the past trends in age-specific mortality. We used the Lee-Carter projection methodology over the use of a multi-population coherent mortality projection methodology because of previous estimates of stagnation in mortality and life expectancy (Islam et al., 2021; Marinetti et al., 2023). also purely considered the past trend in mortality of the country itself.

The expected increase in e30 is based on the assumed continuation of previous mortality improvements, in line with the method used to obtain excess COVID-19 deaths (Islam et al., 2021; Marinetti et al., 2023). For this purpose, we employed, by sex, a Lee-Carter projection of age-specific mortality rates (Lee & Carter, 1992) for the national population and the three educational groups. The Lee-Carter projection is considered a benchmark extrapolative mortality forecasting method (Janssen, 2018), and is also used to obtain excess COVID-19 deaths (Islam et al., 2021; Marinetti et al., 2023).

The first step of the Lee-Carter projection involves the fitting of the Lee-Carter model to historical time series of (logged) age-specific mortality rates.

The formula for the Lee Carter model reads as:

$$log(m_{x,t}) = \alpha_{x}+ \beta_{x}\kappa_{t}+ \mathcal{E}_{x,t}$$

Where $\alpha_{x}$ is the general shape of mortality by age across time, $\kappa_{t}$ is the overall time trend across all ages, and $\beta_{x}$ is the age-specific adjustment of the overall time trend k(t). The second step of the Lee-Carter projection involves the projection of the overall time trend (kt) into the future.

For the start and the end year of the time series for fitting the Lee-Carter model, we selected, to the extent possible, 1999 and 2011, respectively, in line with the outcome of our segmented regression. However, given the relatively large fluctuations in education-specific e30 levels over time, we made some necessary adjustments to the start and the end years for selected educational groups (see Table S2) to make sure that the selected time series truly represented the historical trends. We did so by carefully examining the education-specific historical trends in e30 (see Figure S4).

We performed a sensitivity analysis that used all populations 1972-2011 as the historical time series and 2012-2017 as the projection horizon to obtain expected e30 to add robustness to our decisions regarding the selected years and the impact of using similar years throughout our main results. In this document, in the section titled “Sensitivity analysis regarding the obtainment of expected e30” on page 11, we describe in detailed the results.

To fit the Lee-Carter model, we employed single value decomposition (SVD). For the forecasting, we forecasted the $k_{t}$ terms with a simple random walk with drift using the R package “*forecast*”.

**Table S2.** Information regarding the start and the end year of the historical time-series used for the Lee-Carter forecasting and its forecasting horizon

|  |  | Historical time series | | Forecasting horizon |
| --- | --- | --- | --- | --- |
| Sex | Educational attainment group | Start | End |  |
| Males | National | 1999 | 2011 | 2012-2017 |
|  | Low | 1999 | 2010 | 2011-2017 |
|  | Middle | 1972 | 2000 | 2001-2017 |
|  | High | 1999 | 2011 | 2012-2017 |
| Females | National | 1999 | 2011 | 2012-2017 |
|  | Low | 1999 | 2010 | 2011-2017 |
|  | Middle | 1972 | 2000 | 2001-2017 |
|  | High | 2001 | 2011 | 2012-2017 |

Notes: For middle-educated males and females, we obtained the expected e30 values based on the projection of mortality over the 1972-2000 period. We did this because the e30 values for the middle-educated in 2002-2010 were substantially lower than the historical trend (see Figure S4 in the Supplementary File1), likely as a result of data issues in the 2001-2011 follow-up (see Janssen et al. 2024).

We used 2010 as the end year of the historical time series for the low-educated, given that the stagnation had already started in 2010 among low-educated males.

We used 2001 instead of 1999 or 2000 as the start year for high-educated females because 1999-2000 represented outliers (see Figure S4 in the Supplementary File1).

**Figure S4.** Observed (1971-2017)* and expected** (2011-2017) trends in remaining life expectancy at age 30 (e30), by sex, in England & Wales. A) At the national level and B) by educational attainment group. *The vertical dashed lines represent the start of the stagnation in e30.*


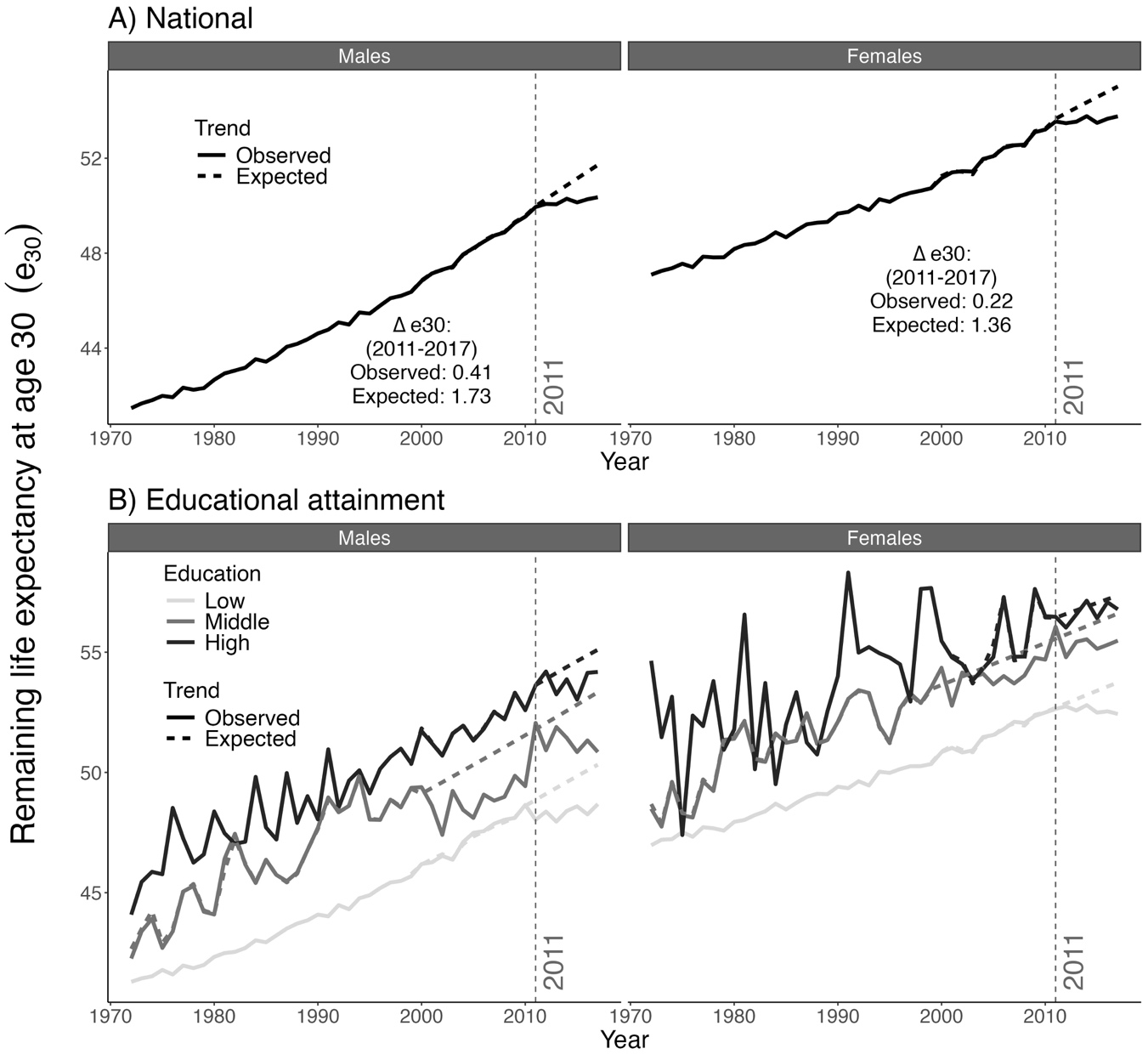


* For middle-educated males and females, we obtained the expected e30 values based on the projection of mortality over the 1972-2000 period. We did this because the e30 values for the middle-educated in 2002-2010 were substantially lower than the historical trend, likely as a result of data issues in the 2001-2011 follow-up (see Janssen et al. 2024).

** We used Lee-Carter model to obtain expected e30 trends based on the period 1990 to 2017. Only, for middle-educated males and females, we obtained the expected e30 values based on the projection of mortality over the 1972-2000 period.

Source data: ONS Longitudinal Study.

- Decomposition method

To assess the contribution of each educational group to the e30 stagnation, we decomposed, by sex, the difference between the observed and the expected changes in e30 over the 2011-2017 period. For this purpose, we extended the stepwise replacement decomposition method (Andreev et al., 2002) and used the R package “*DemoDecomp*” (Riffe, 2014). Our extension of the stepwise replacement decomposition method assumes that the stagnation in e30 is the net result of the expected and the observed trend and their underlying changes in the age-specific mortality rates by educational group, weighted by the changes in population composition.

- Assessing the contribution of increasing educational inequalities to e30 stagnation

In an additional analysis, we assessed the contribution of educational inequalities in remaining life expectancy at age 30 (e30) to the overall national life expectancy stagnation in e30. We did so by comparing the observed stagnation with the stagnation if educational inequalities in e30 during 2011-2017 would have remained constant at the average level for 2000-2010.

First, we computed the absolute educational inequalities in e30 between the high-educated and low-educated and between the high-educated and the middle-educated by year (2000-2017) and sex.

Second, we computed the average level of educational inequalities in e30 in the period 2000-2011 by sex. The average level of educational inequalities between high and low-educated was 4.63 years for males and 3.66 years for females, while the average level of educational inequalities between high and middle-educated was 1.54 years for males and 0.73 years for females.

Third, we estimated the e30 levels for the low and middle-educated for the period 2011-2017 assuming constant educational inequalities in e30, by applying the abovementioned average inequality levels to the e30 values for the high-educated for the years 2011-2017.

For the results section, we used the observed e30 for high education because it captures the impact of the observed inequalities on the e30 stagnation, see Table S1 in the Supplementary File 2.

Fourth, taking the weighted average of the obtained e30 levels by educational level from the previous step, we obtained an approximation of the new national e30.

Fifth, we compared the observed stagnation (expected minus observed change in e30 in 2011-2017) with the stagnation under constant inequalities (expected change in e30 minus change in e30 assuming constant inequality).

Finally, we obtained an approximation of the contribution of educational inequalities in e30 to the overall national life expectancy stagnation in e30, by dividing the observed stagnation minus the stagnation under constant inequalities, with the observed stagnation.

- Sensitivity analysis regarding the obtainment of expected e30

To add robustness to our decisions regarding the selected years and the impact of using similar years throughout our main results, we performed a sensitivity analysis that used for all populations 1972-2011 as the historical time series and 2012-2017 as the projection horizon to obtain expected e30.

Figure S5 shows the time trends in observed and expected e30 trends by the period of reference used for the forecast (2011-2017), we observed that at the national level, as expected the differences increased over time, but still the relative differences between the expected e30 using the long trends or the shorts is less than 1% (See Supplementary Figure S5). The results by educational attainment groups portray mixed results. For low-educated groups, for males, we observed large differences, but that is because of the differences in the year of the start of the forecasting, while for females we observed very small differences. For middle-educated males, we do not observe differences, while for middle-educated females the long-term trends would lead to a higher e30. For high-educated groups, for males, the short series led to a higher e30, but the differences with the expected e30 using the long-term series is 0.03%, while for females, we observed the largest difference 1%, and the short-series estimate a higher e30.

In this sensitivity analysis, we estimated a stagnation of e30 at the national level of 1.02 years (males) and 0.79 years (females) – that is, 0.30 years (males) and 0.35 years (females) less than in the main analysis – and a pattern similar to that in the main analysis for the contribution of education to e30 stagnation. Looking at trends by educational group, we observed that the level of stagnation was high among low-educated females, while it was lower among other low-educated groups (See Figure S6). That is because we started the forecast in 2011 in the main analysis, as we observed an early year of stagnation among low-educated groups.

Nonetheless, the decomposition of the stagnation of e30 for this sensitivity analysis (Figure S7) revealed largely similar outcomes. Specifically, we found that the low-educated still contributed the most to the stagnation (85% instead of 86%) among females and that the low-educated (22% instead of 41%) and the middle-educated (66% instead of 54%) still contributed the most to the stagnation among males.

**Figure S5.** Time trends in observed (1972-2017) and expected (2011-2017) remaining life expectancy at age 30 (e30), by sex, in England & Wales, thereby comparing the use of the baseline period for the Lee-Carter projection in our main analysis (1999-2011 with some exceptions*) with a longer baseline period for the Lee-Carter projection in our sensitivity analysis (1972-2011 throughout) to obtain expected e30.


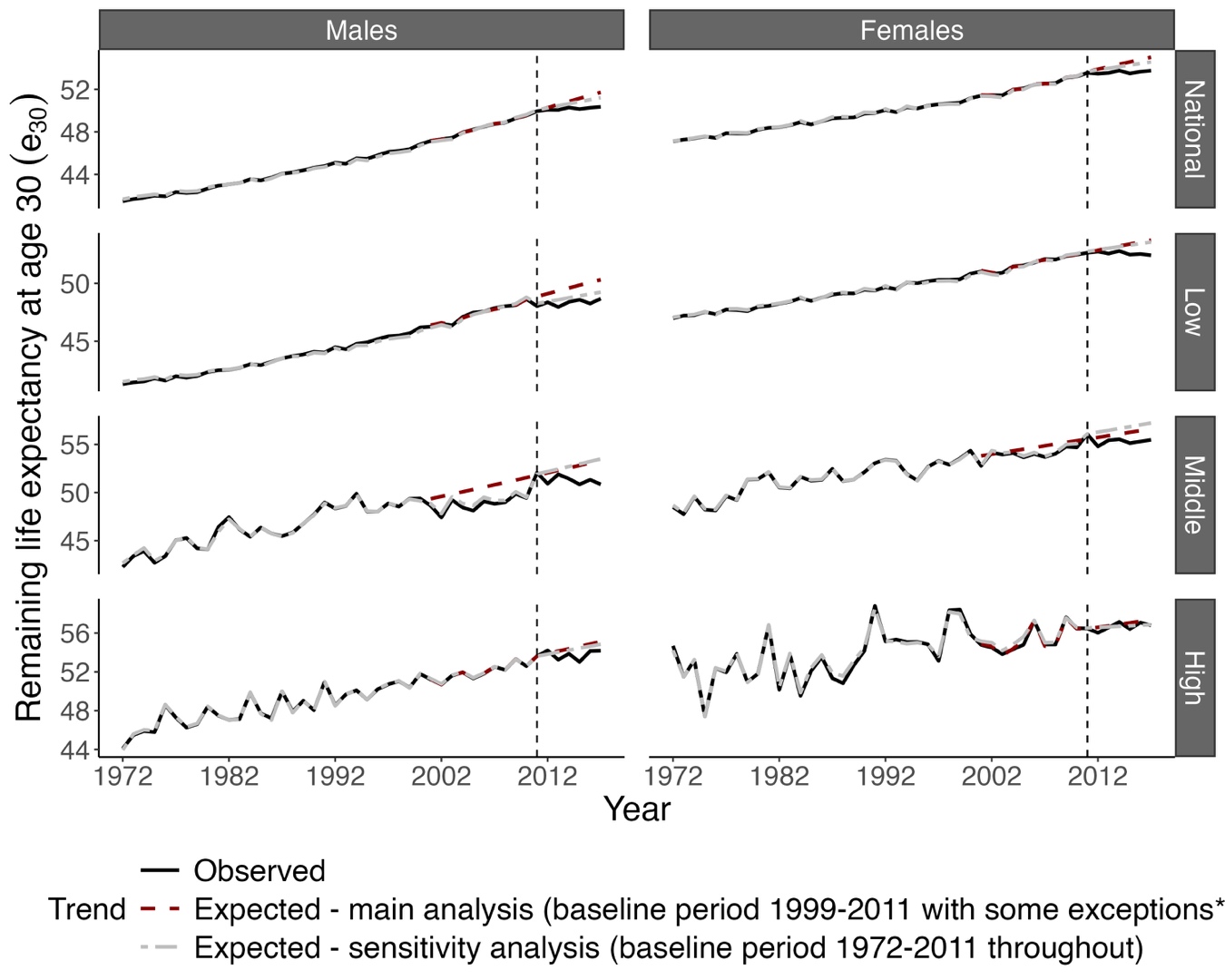
* For middle-educated males and females, we obtained the expected e30 values based on the projection of mortality over the 1972-2000 period.

Source data: ONS-Longitudinal Study

**Figure S6.** Time trends in observed (1972-2011) and expected (2012-2017) remaining life expectancy at age 30 (e30) at the national level and for the different educational groups based on our sensitivity analysis in which we used the trends from 1972-2011 to obtain the expected e30 for 2012-2017. England & Wales.

**
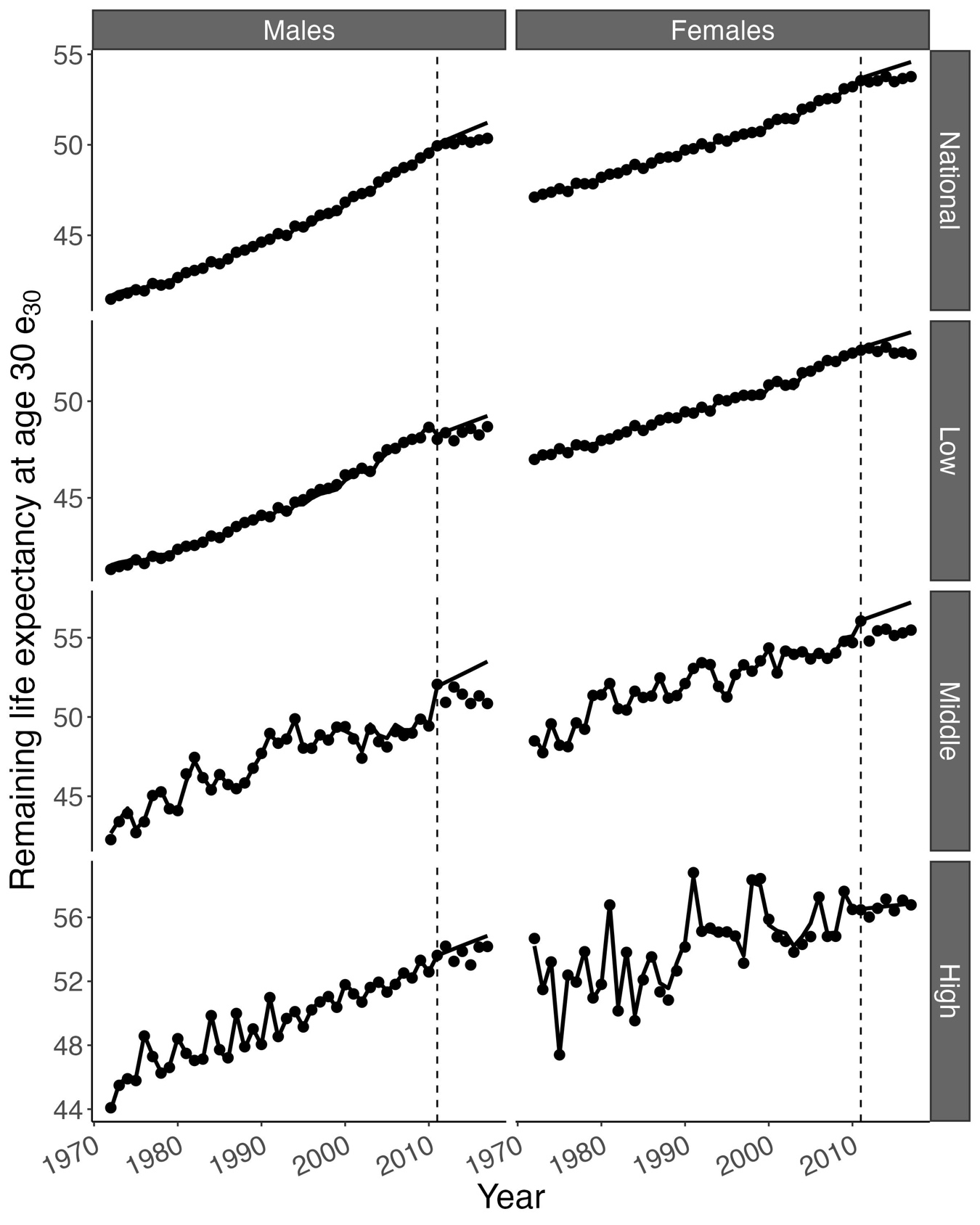
**

Source data: ONS Longitudinal Study.

**Figure S7.** Contribution of education-specific mortality trends to the observed stagnation in e30 from 2011-2017 (1.02 years among males; 0.79 years among females), by sex, England & Wales. Results from our sensitivity analysis in which we used the trends from 1972-2011 throughout to obtain the projection for 2012-2017.

**
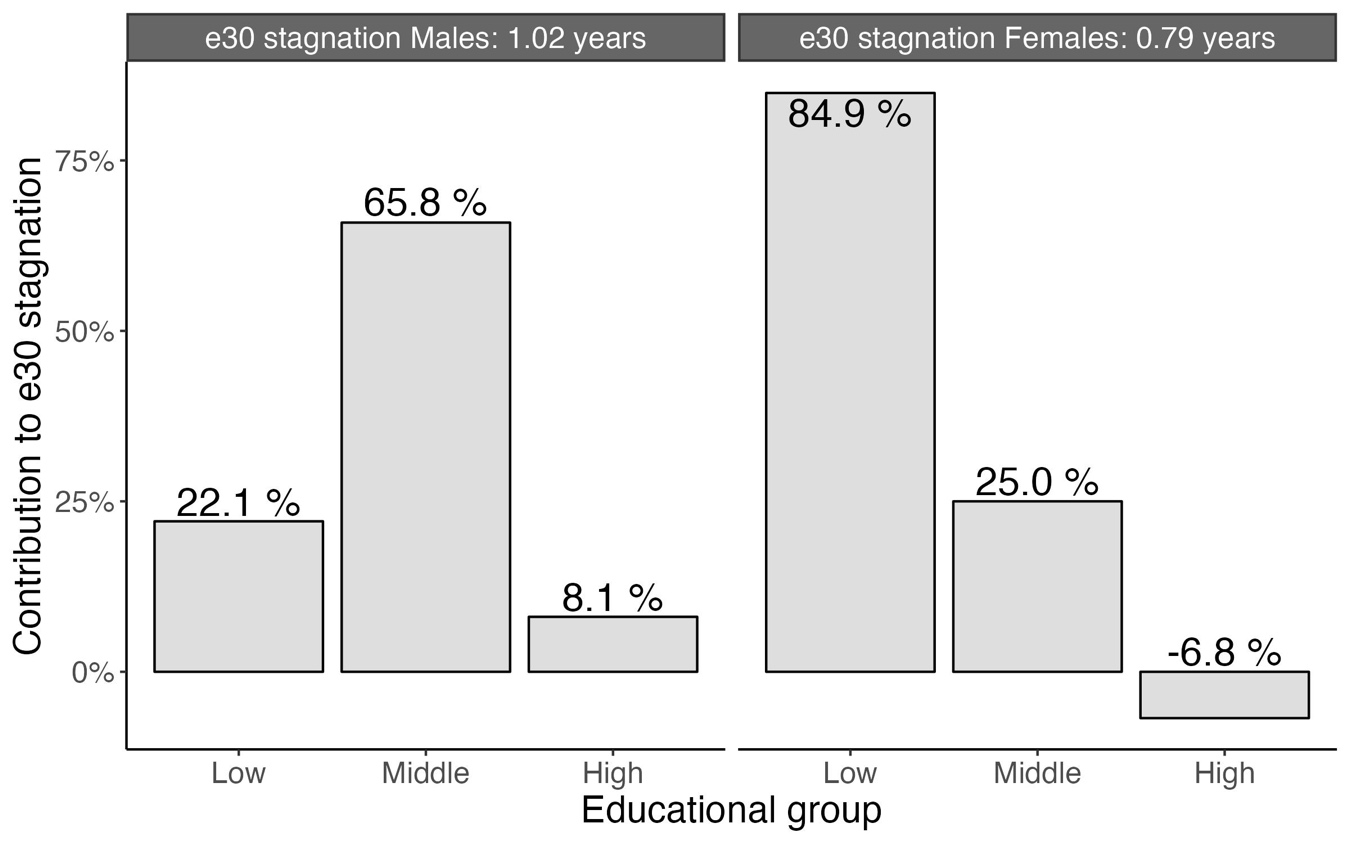
**

*The abovementioned contributions add up to 104% for males and 103% for females, because the contributions were counterbalanced by 3.92% among males and 3.09% among females by changes over time in the population composition by education.

Source data: ONS Longitudinal Study

References

Aburto, J. M., Schöley, J., Kashnitsky, I., Zhang, L., Rahal, C., Missov, T. I., Mills, M. C., Dowd, J. B., & Kashyap, R. (2021). Quantifying impacts of the COVID-19 pandemic through life-expectancy losses: a population-level study of 29 countries. *International Journal of Epidemiology*, *51*(1), 63-74. https://doi.org/10.1093/ije/dyab207

Abrams, LR. M. Myrskylä, N.K. Mehta (2023). The “double jeopardy” of midlife and old age mortality trends in the United States, *Proc. Natl. Acad. Sci. U.S.A.* 120 (42) e2308360120. https://doi.org/10.1073/pnas.2308360120

Andreev, E. M., Shkolnikov, V., & Begun, A. (2002). Algorithm for decomposition of differences between aggregate demographic measures and its application to life expectancies, healthy life expectancies, parity-progression ratios and total fertility rates. *Demographic Research*, *7*(14), 499-522. https://www.demographic-research.org/volumes/vol7/14/

de Gelder, R., Menvielle, G., Costa, G., Kovács, K., Martikainen, P., Strand, B. H., & Mackenbach, J. P. (2017). Long-term trends of inequalities in mortality in 6 European countries. *International Journal of Public Health*, *62*(1), 127-141. https://doi.org/10.1007/s00038-016-0922-9

Eikemo, T. A., Hoffmann, R., Kulik, M. C., Kulhánová, I., Toch-Marquardt, M., Menvielle, G., Looman, C., Jasilionis, D., Martikainen, P., Lundberg, O., Mackenbach, J. P., & for the, E.-G. B. D. S. E. c. (2014). How Can Inequalities in Mortality Be Reduced? A Quantitative Analysis of 6 Risk Factors in 21 European Populations. *PLOS ONE*, *9*(11), e110952. https://doi.org/10.1371/journal.pone.0110952

HMD. (2021). *Human Mortality Database, Max Planck Institute for Demographic Research (Germany), University of California, Berkeley (USA), and French Institute for Demographic Studies (France). Retrieved November 19, 2021*. Available at www.mortality.org

Islam, N., García López, F. J., Jdanov, D. A., Royo- Bordonada, M. Á., Khunti, K., Lewington, S., Lacey, B., White, M., Morris, E. J., & Zunzunegui, M. V. (2021). Unequal impact of the Covid-19 pandemic on excess deaths, life expectancy, and premature mortality across Spanish regions in 2020 and 2021. *medRxiv*, 2021.2011.2029.21266617. https://doi.org/10.1101/2021.11.29.21266617

Islam, N., Shkolnikov, V. M., Acosta, R. J., Klimkin, I., Kawachi, I., Irizarry, R. A., Alicandro, G., Khunti, K., Yates, T., Jdanov, D. A., White, M., Lewington, S., & Lacey, B. (2021). Excess deaths associated with covid-19 pandemic in 2020: age and sex disaggregated time series analysis in 29 high income countries. *BMJ*, *373*, n1137. https://doi.org/10.1136/bmj.n1137

Janssen, F. (2018). Advances in mortality forecasting: introduction. *Genus*, *74*(1), 21. https://doi.org/10.1186/s41118-018-0045-7

Janssen, F., Van Hemelrijck, W., Kagenaar, E., & Sizer, A. (2024). Enabling the examination of long-term mortality trends by educational level for England and Wales in a time-consistent and internationally comparable manner. *Population Health Metrics*, *22*(1), 4. https://doi.org/10.1186/s12963-024-00324-2

Lee, R. D., & Carter, L. R. (1992). Modeling and forecasting US mortality. *Journal of the American Statistical Association*, *87*(419), 659--671. https://doi.org/10.2307/2290201

Marinetti, I., Jdanov, D., Grigoriev, P., Klüsener, S., & Janssen, F. (2023). Effects of the COVID-19 pandemic on life expectancy and premature mortality in the German federal states in 2020 and 2021. *PLOS ONE*, *18*(12), e0295763. https://doi.org/10.1371/journal.pone.0295763

Muggeo, V. M. (2008). Segmented: an R package to fit regression models with broken-line relationships. *R news 8*, 20-25.

ONS. (2019). *ONS Longitudinal Study – England and Wales. Office for National Statistics, Released 11 June 2019,* ONS SRS Metadata Catalogue. https://doi.org/doi: 10.57906/z9xn-ng05

Preston, S., Heuveline, P., & Guillot, M. (2001). *Demography: measuring and modeling population processes*. Blackwell Publishers.

Riffe, T. (2014). *Package ‘DemoDecomp’. Decompose Demographic Functions*. In (Version R package version 1.0. 1)

Shelton, N., Marshall, C. E., Stuchbury, R., Grundy, E., Dennett, A., Tomlinson, J., Duke-Williams, O., & Xun, W. (2018). Cohort Profile: the Office for National Statistics Longitudinal Study (The LS). *International Journal of Epidemiology*, *48*(2), 383-384g. https://doi.org/10.1093/ije/dyy243

Silcocks, P. B. S., Jenner, D. A., & Reza, R. (2001). Life expectancy as a summary of mortality in a population: statistical considerations and suitability for use by health authorities. *Journal of Epidemiology and Community Health*, *55*(1), 38-43. https://doi.org/10.1136/jech.55.1.38

UNESCO. (1997). *International standard classification of education-ISCED 1997*. United Nations Educational, Scientific and Cultural Organization.
