# Supplementary material for: The contribution of education-specific mortality trends to the life expectancy stagnation in England & Wales
Source: Eur J Epidemiol. 2025 Jun 2;40(5):511–5. doi: 10.1007/s10654-025-01251-8 (PMC12170670; doi:10.1007/s10654-025-01251-8)
Supplement: Supplementary file 2 — Supplementary Material 2 [file 10654_2025_1251_MOESM2_ESM.docx]

Supplementary File 2 –

The Contribution of Education-Specific mortality trends to the life expectancy stagnation in England & Wales

SUPPLEMENTARY TABLES AND FIGURES

**Journal:** European Journal of Epidemiology

**Table S1.** Contribution of educational inequalities to overall stagnation in remaining life expectancy at age 30 (e30), by sex, in England & Wales (2011-2017). *The Constant inequality obs. scenario indicates if educational inequalities in e30 during 2011-2017 would have remained constant at the average level for 2000-2010.*

| Trend | Increases in e30 (2011-207) | Stagnation* | Estimated contribution of educational inequalities to the stagnation** |
| --- | --- | --- | --- |
| Males | | | |
| Observed | 0.41 | 1.32 |  |
| Expected | 1.73 |  |  |
| Constant inequality | 0.76 | 0.97 | 26.5 |
| Females | | | |
| Observed | 0.22 | 1.14 |  |
| Expected | 1.36 |  |  |
| Constant inequality | 0.53 | 0.83 | 27.2 |

* Expected increases in e30 minus the observed increases in e30 or minus the increases in e30 when assuming constant inequality.

** Observed stagnation minus stagnation using constant inequality, divided by observed stagnation, times 100.

Source data: ONS Longitudinal Study.

**Table S2.** Overall and annual change in life expectancy at birth (e0) and in remaining life expectancy at age 30 (e30), by sex, in England & Wales (1999-2017)

| Sex | Change in life expectancy (in years) | | Annual pace of change in life expectancy | | | |
| --- | --- | --- | --- | --- | --- | --- |
|  |  |  | Years | | Months | |
|  | 1999-2011 | 2011-17 | 1999-2011 | 2011-17 | 1999-2011 | 2011-17 |
| Changes in life expectancy at birth (e0) | | | | | | |
| Males | 3.90 | 0.50 | 0.30 | 0.07 | 3.60 | 0.84 |
| Females | 2.99 | 0.26 | 0.23 | 0.04 | 2.76 | 0.48 |
| Changes in remaining life expectancy at age 30 (e30) | | | | | | |
| Males | 3.58 | 0.41 | 0.28 | 0.06 | 3.36 | 0.72 |
| Females | 2.81 | 0.22 | 0.22 | 0.03 | 2.64 | 0.36 |

Source data: ONS Longitudinal Study and the Human Mortality Database

**Figure S1.** Annual stagnation* in e30 (2011-2017) (in months) after the start of stagnation for the national population and the different educational groups, by sex, in England & Wales.


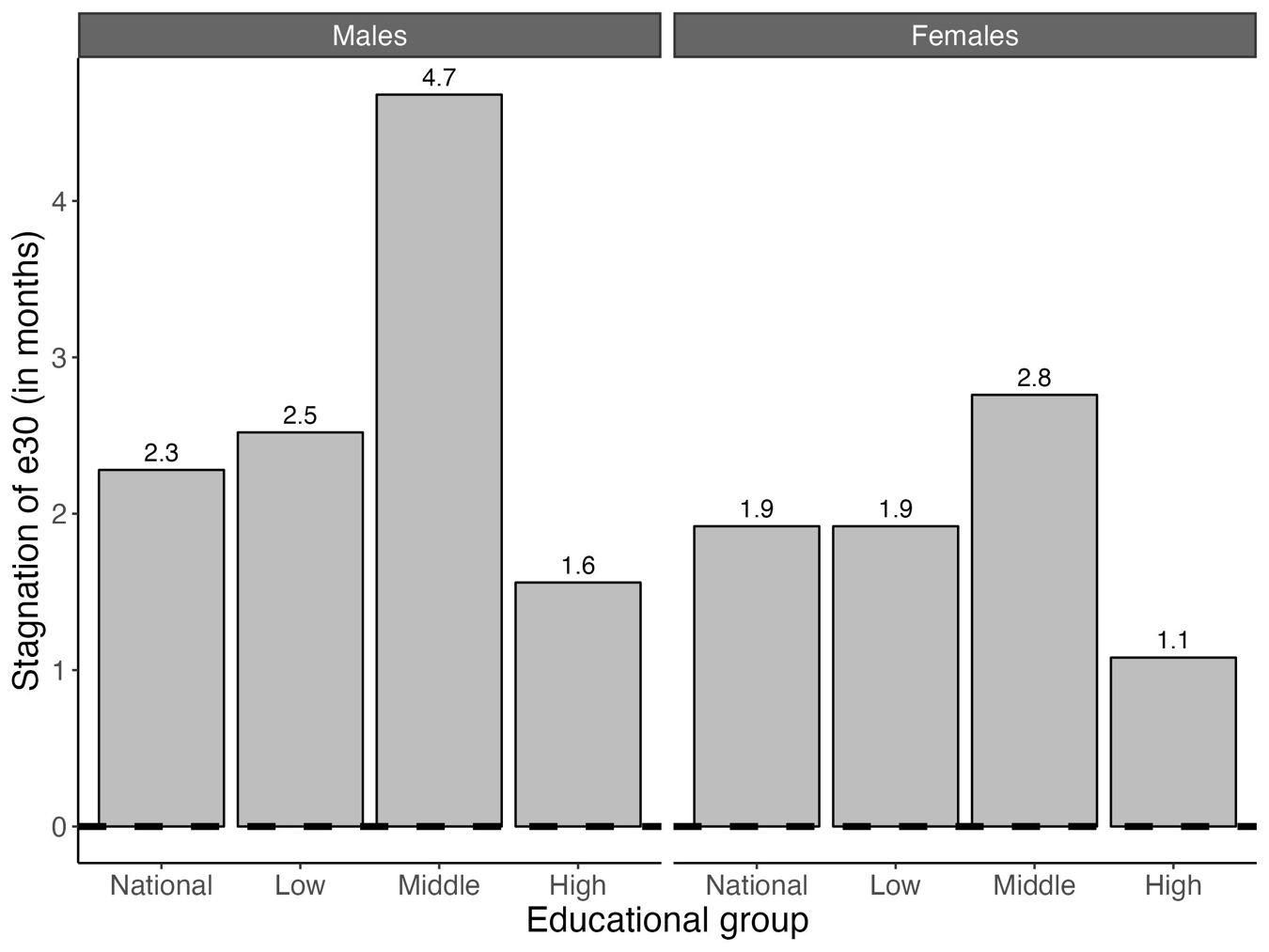


*Annual stagnation in months is measured as the transformation of the annual stagnation into months, where stagnation is the difference between expected increases in e30 between 2011-2017 minus observed increases in e30 between 2011-2017.

Source data: ONS Longitudinal Study

**Figure S2.** Trends over time in the share of the population by educational attainment group (%), for those aged 30 and older, by sex. England & Wales (1972-2017). *The vertical dashed line represents the beginning of the study period 1999-2017.*


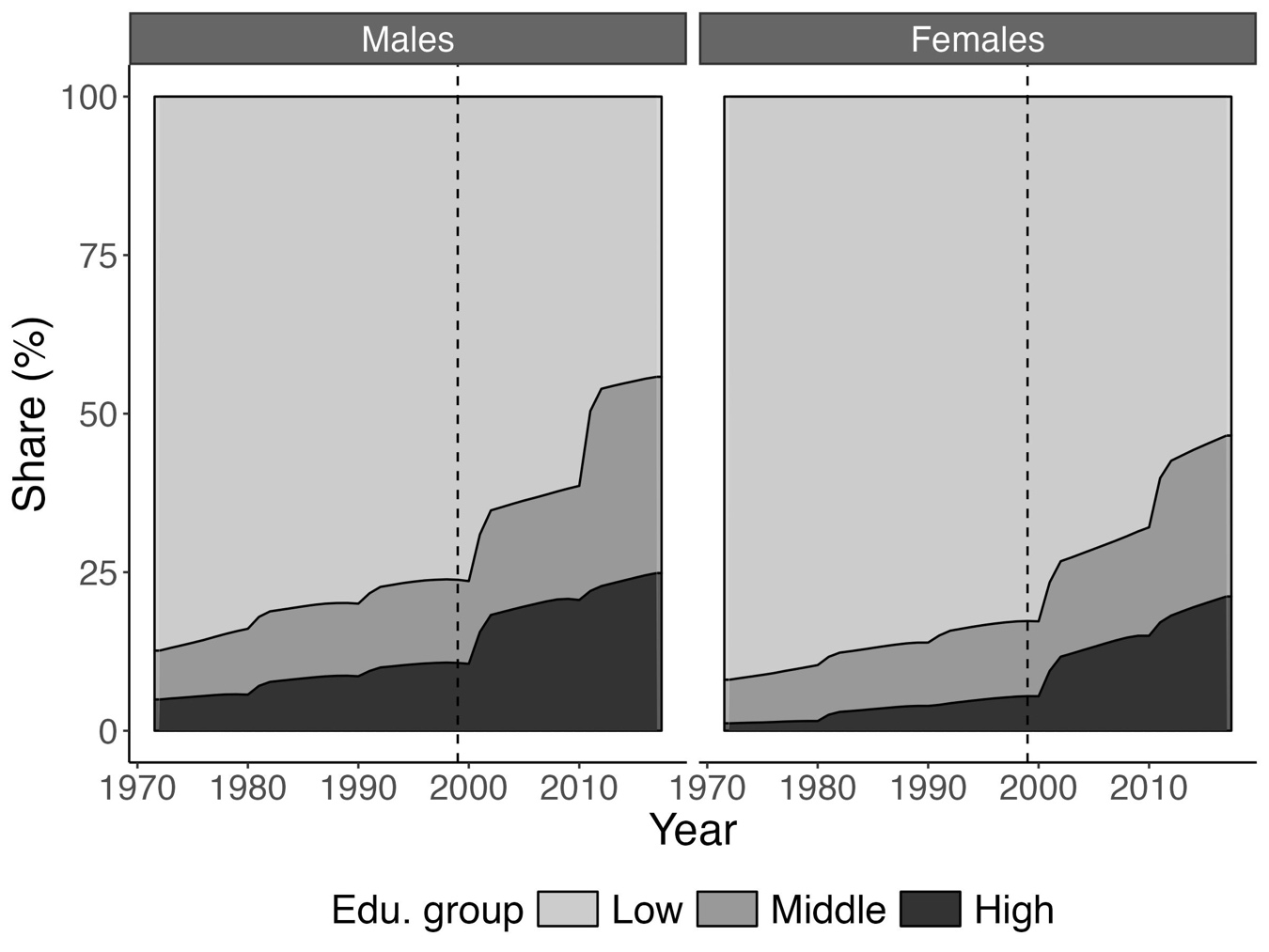


Source data: ONS-Longitudinal Study

**Figure S3.** Educational inequalities in remaining life expectancy at age 30 (e30) (e30 high educated minus e30 low educated (in years)), by sex, England & Wales, 2000-2017, including the average inequality before the stagnation (=for 2000-2010) (dotted trend line). *See Supplementary File 1 section “Assessing the contribution of increases in educational inequalities in e30 to national e30 stagnation” for more information. The vertical dashed line represents the start of national e30 stagnation in 2011.*


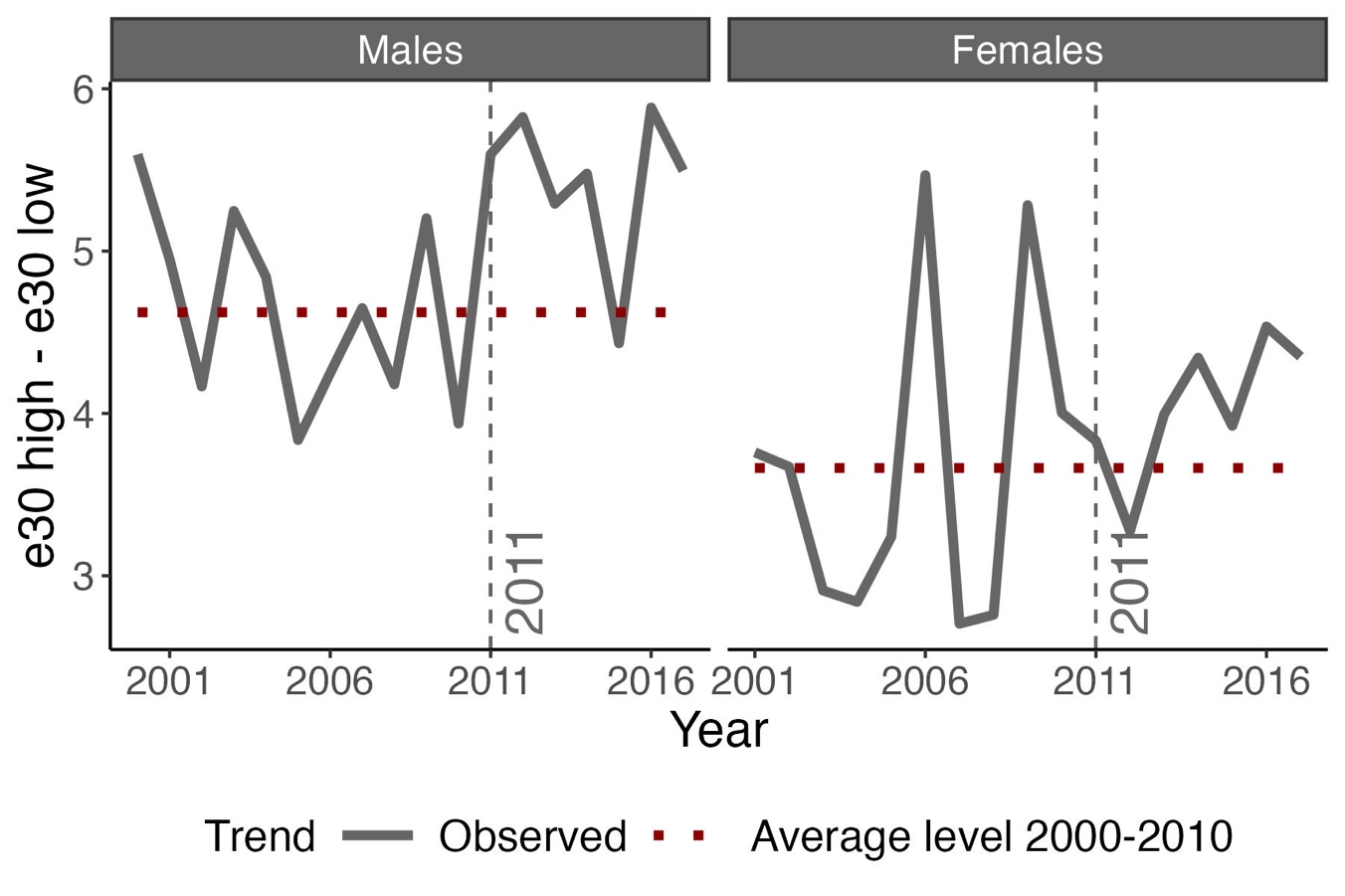


Source data: ONS-Longitudinal Study

**Figure S4.** Increases in remaining life expectancy at age 30 (e30) for England & Wales in 2011-2017, observed versus expected versus using a constant educational inequality in e30, by sex. *See Supplementary File 1 section “Assessing the contribution of increases in educational inequalities in e30 to national e30 stagnation” for detailed information on this analysis.*


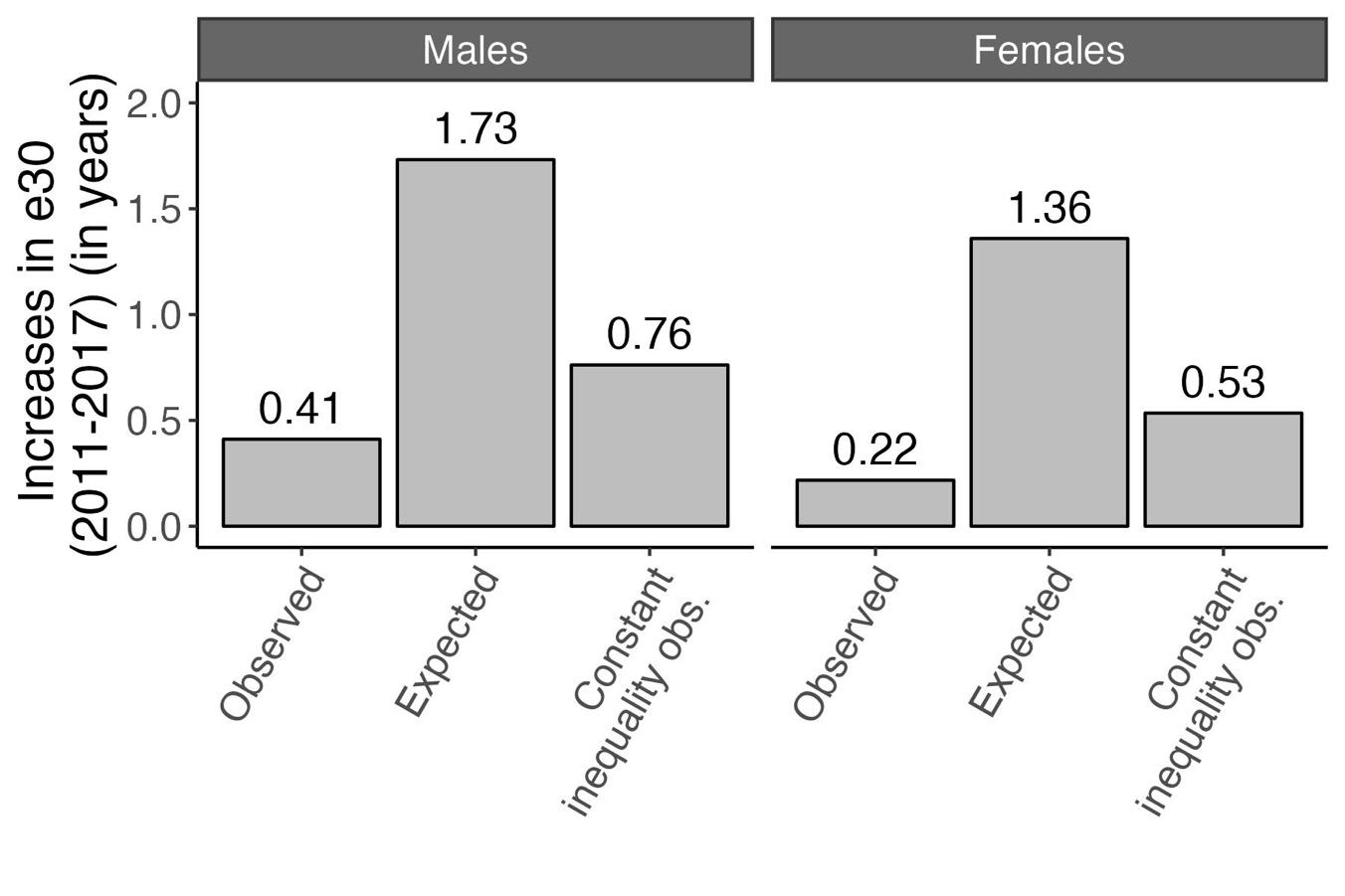
Source data: ONS-Longitudinal Study

**Constant inequality obs., indicates that we used observed trends in e30 of high-educated to create the constant inequality scenarios. Constant inequality exp. indicates that we used expected trends in e30 of high-educated to create the constant inequality scenarios.*

**Figure S5.** Time trends in life expectancy at birth (e0) and in remaining life expectancy at age 30 (e30), by sex, in England & Wales, 1999-2017. The vertical dashed line represents the start of stagnation in the increase in e0 and e30.

**
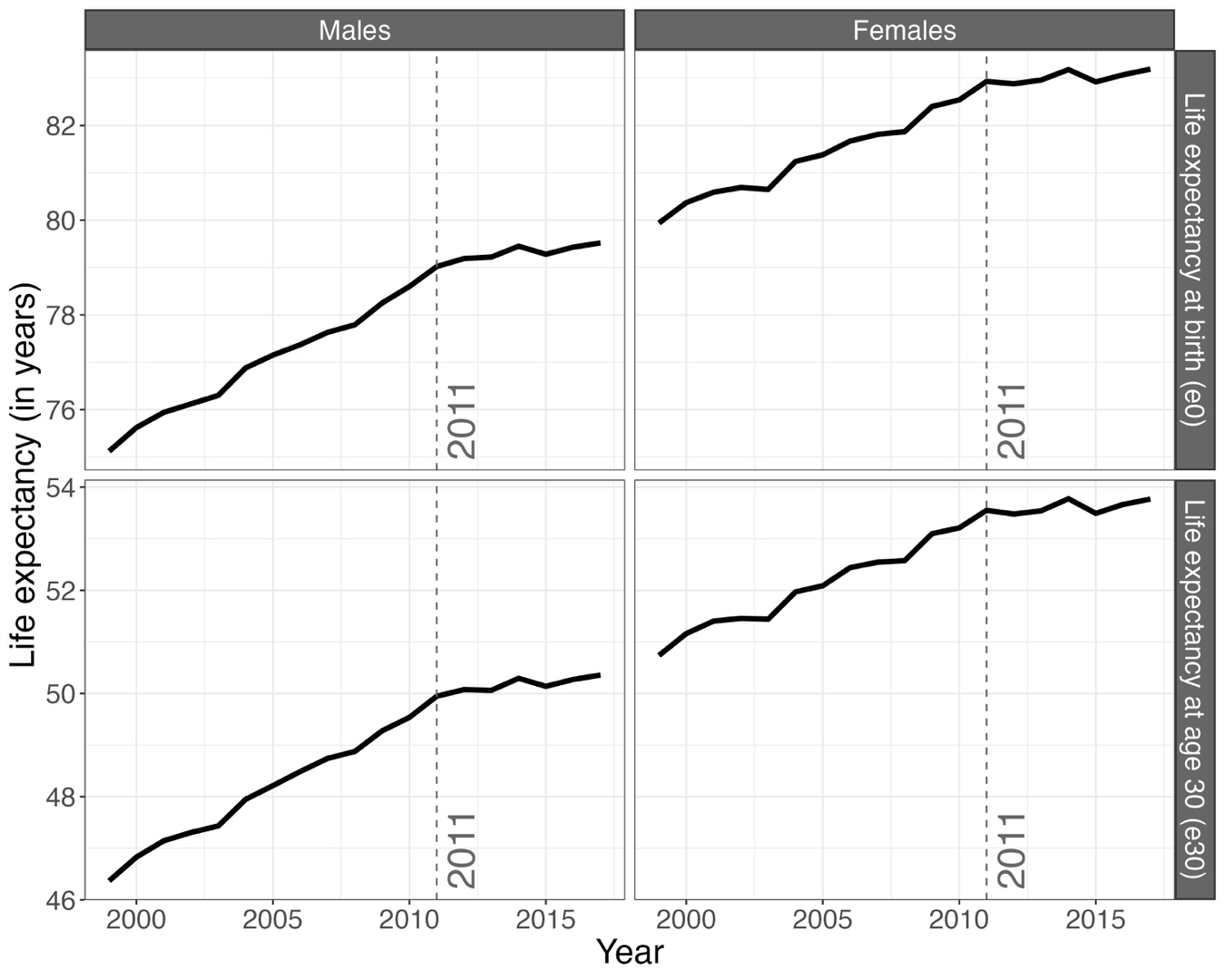
**

Source data: ONS Longitudinal Study and the Human Mortality Database

**Figure S6.** Contribution of mortality by different age groups to changes in remaining life expectancy at age 30 (e30) in England & Wales before* and after** the stagnation in 2011, by sex and educational attainment group


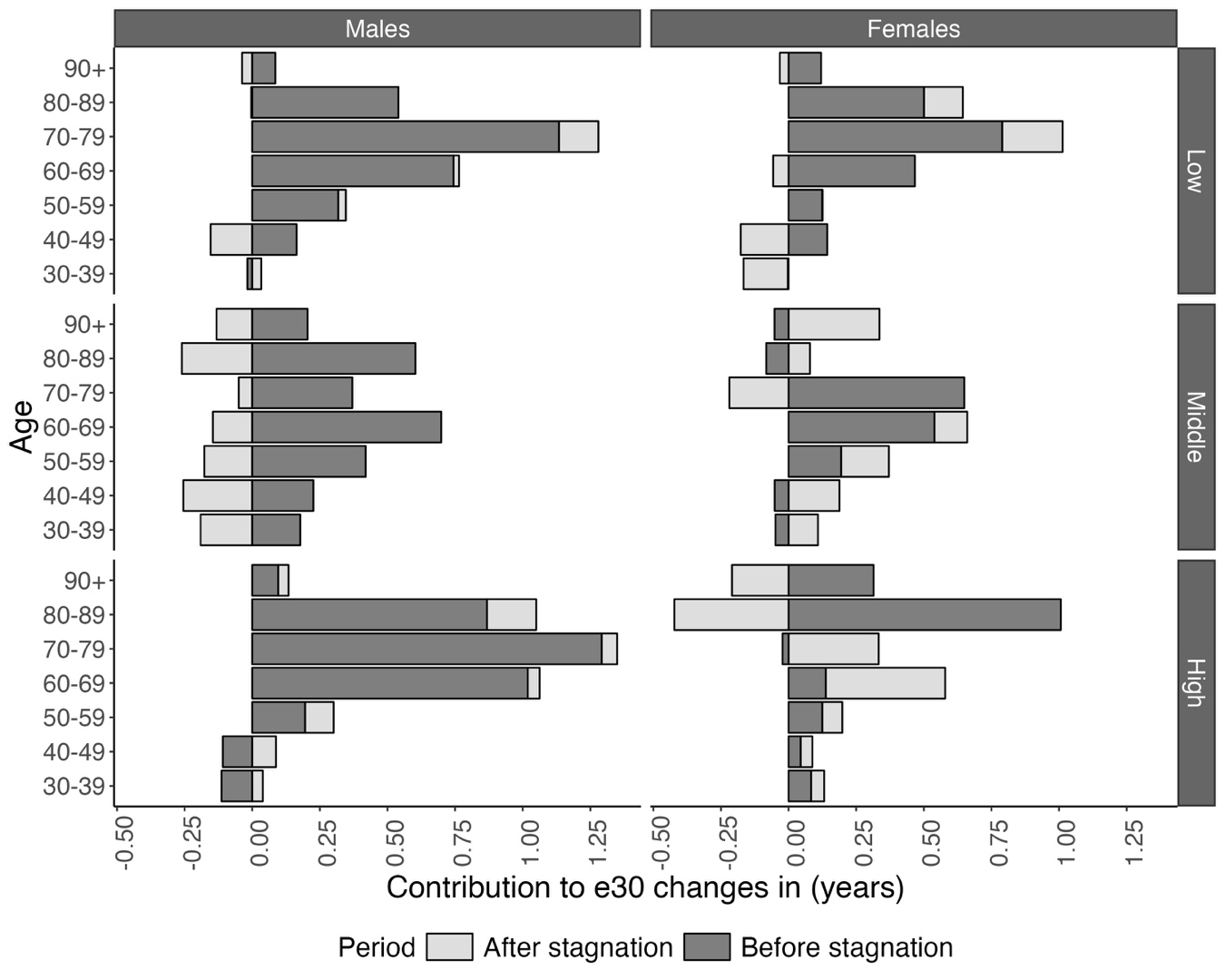


* Before stagnation covers the change in e30 between 2011 and 1999, except for high-educated females (2001-2011), given that the e30 value for 1999 was an outlier; and for low-educated groups (1999-2010), given that the e30 value for 2010 was higher and declined in 2011. For a detailed explanation, see Table S2 in the Supplementary File 1).

** After stagnation covers the change in e30 between 2017 and 2011.

Source data: ONS Longitudinal Study

**Figure S7.** Total excess deaths (= observed deaths minus expected deaths) over the 2011-2017 period due to the observed stagnation in e30 in 2011 in England & Wales, by sex and educational group


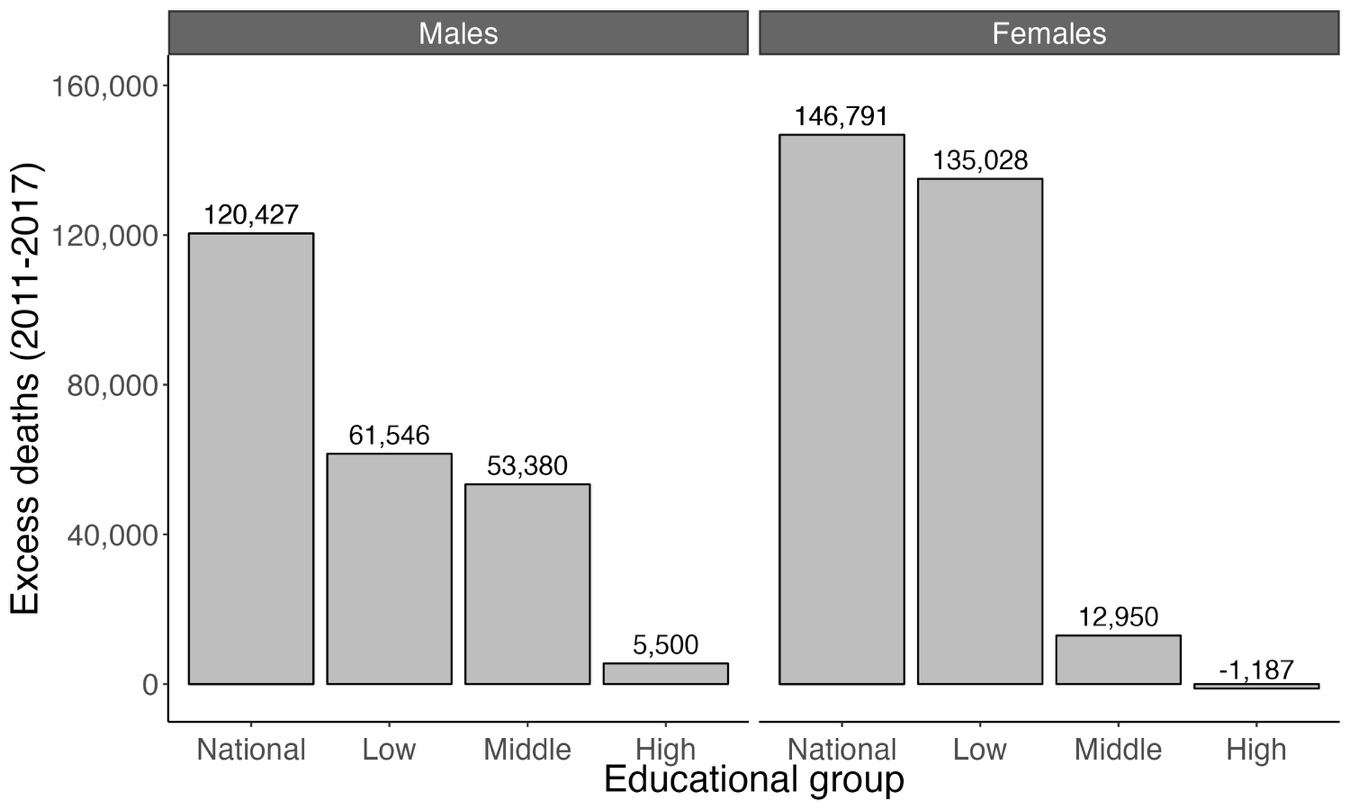


Source data: ONS Longitudinal Study
